# Headache associated with adverse cognitive trajectories in Chinese aging cohort: a group-based trajectory modeling study

**DOI:** 10.1007/s10072-026-08922-8

**Published:** 2026-03-07

**Authors:** Hong Wang, Weisheng Deng, Wei Qiu

**Affiliations:** Department of Neurology, Meizhou People’s Hospital, Meizhou, Guangdong Province China

**Keywords:** ​Headache​, ​Cognitive trajectories, Group-based trajectory modeling, ​Alcohol, ​Hypertension​, China Health and Retirement Longitudinal Study

## Abstract

**Background:**

Conflicting evidence exists regarding the associations between headache- and cognition, with conventional methods failing to capture the heterogeneous cognitive trajectories. We investigated dynamic relationships using group-based trajectory modeling (GBTM).

**Methods:**

This longitudinal analysis included 2,949 participants aged ≥45 from the China Health and Retirement Longitudinal Study (CHARLS, 2011-2020). Headache was ascertained via a standardized questionnaire. Cognitive trajectories were derived from global cognition Z-scores using GBTM. Multinomial logistic regression evaluated headache-cognition associations, adjusting for demographics, health behaviors, and comorbidities. Stratified analyses tested effect modification by demographics, health behaviors, and comorbidities

**Results:**

GBTM identified three cognitive trajectories: low (17.5%), medium (43.8%), and high (38.7%). Headache significantly increased the probability of belonging to low (OR = 1.29, 95% CI: 1.11–1.50; p < 0.001) and medium trajectories (OR = 1.14, 95% CI: 1.01–1.30; p = 0.033). Alcohol, hypertension, and stroke amplified associations, while diabetes paradoxically attenuated them (p for interaction < 0.05).

**Conclusion:**

Headache independently predicts adverse cognitive trajectories, with alcohol, hypertension, and stroke acting as critical effect modifiers. Paradoxically, diabetes attenuated this risk. Integrating headache screening into cognitive risk stratification and targeting modifiable factors may mitigate cognitive risk.

**Supplementary Information:**

The online version contains supplementary material available at 10.1007/s10072-026-08922-8.

## Introduction

Headache disorders demonstrate a global high prevalence, with significantly rising incidence among aging populations [1–3]. Substantial evidence links headaches to cognitive decline and dementia risk [3–5], mediated through neuroinflammatory pathways and white matter lesions [6–8]. Accelerated population aging further exacerbates the socioeconomic burden of headache-related cognitive impairments [9, 10].

Current evidence remains conflicting: while some cohorts report no significant migraine-dementia association [9, 11–13], others confirm elevated cognitive impairment risk in headache patients [3, 5, 14]. Critical methodological limitations persist—conventional approaches like linear mixed models capture individual variances but fail to identify population-heterogeneous cognitive trajectory subtypes ​​ [15]. Although group-based trajectory modeling (GBTM) resolves this gap by characterizing developmental heterogeneity [16], its application in headache-cognition dynamics remains unexplored.

To address this gap, we leverage repeated cognitive measurements from the nationally representative China Health and Retirement Longitudinal Study (CHARLS) cohort. Using GBTM, we systematically investigate dynamic associations between headache exposure and specific cognitive trajectory patterns. We hypothesize that individuals with headaches will demonstrate significantly higher probability of following adverse cognitive trajectories (e.g., low or medium cognition) than their headache-free counterparts. These findings will advance epidemiological evidence for precision headache management interventions to mitigate cognitive decline.

## Methods

### Data source​

This study utilized data from the CHARLS, a nationally representative longitudinal cohort initiated in 2011, targeting Chinese adults aged 45 years and above. CHARLS employs multistage stratified probability-proportional-to-size sampling across 150 counties/districts in 28 provinces, achieving an 80.5% baseline response rate to minimize selection bias [17]. The study conducts biennial follow-ups with five completed waves by 2020 (http://charls.pku.edu.cn/), providing high-quality data on aging populations’ health, economic, and social conditions. Ethical approval was granted by the Peking University Biomedical Ethics Committee, with written informed consent obtained from all participants.

### Study population

**Fig. 1:**
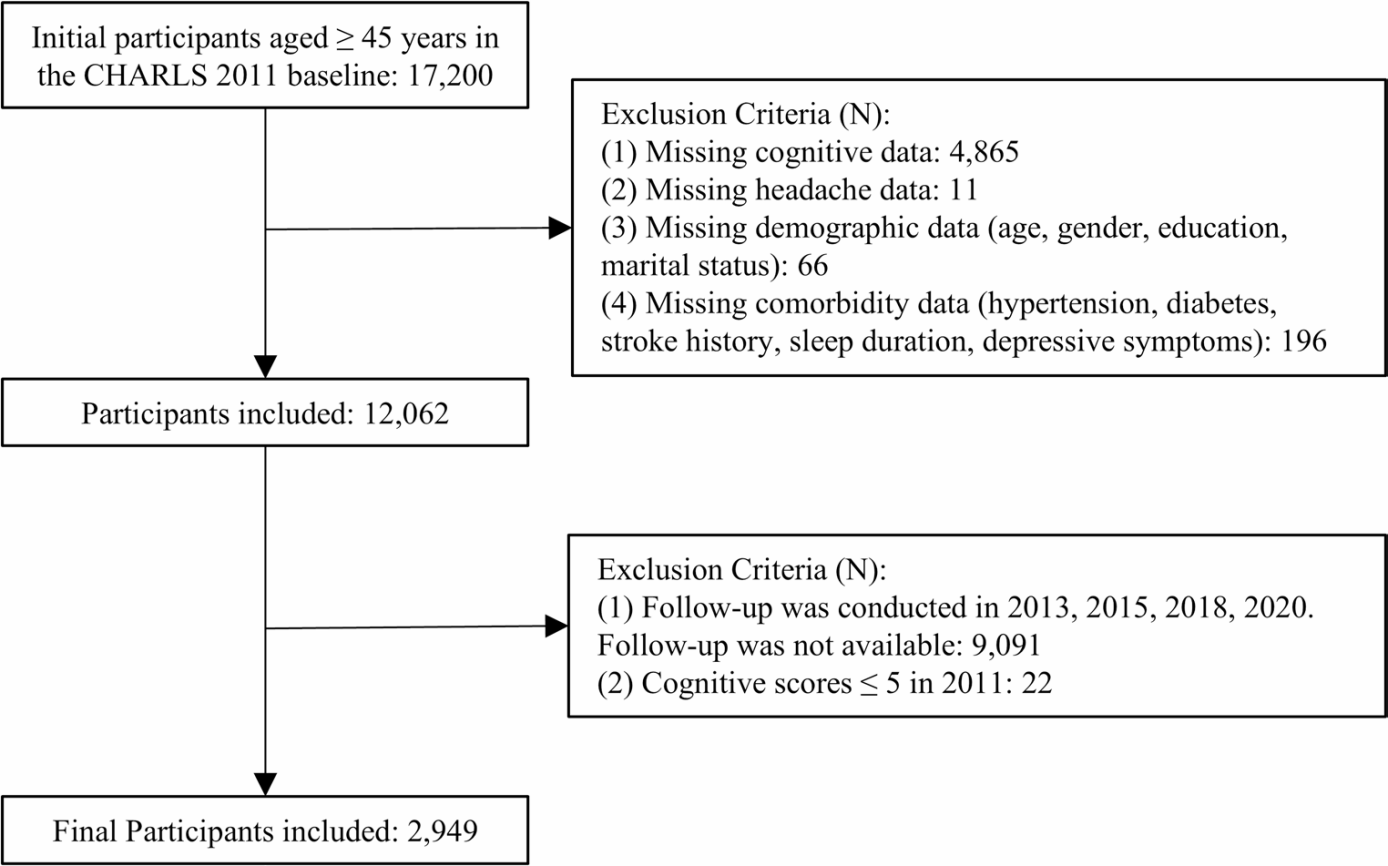
Participant inclusion flowchart of the CHARLS cohort study

The analytical sample comprised 17,200 individuals aged ≥ 45 from the CHARLS Wave 1 baseline survey (2011). After rigorous screening, including the exclusion of 22 individuals with baseline cognitive impairment (defined as a cognitive score ≤ 5, 1.5 SD below the mean) to address potential reverse causality [18], 2,949 eligible participants were retained with complete baseline data and longitudinal cognitive assessments across all five waves (2011–2020). Figure 1 details the selection flowchart.

### Headache assessment

Headache status was ascertained through a standardized questionnaire item asking participants to report the presence of head pain symptoms. Affirmative responses classified individuals as headache-positive, while negative responses denoted headache-negative status, establishing a dichotomous exposure variable.

### Cognitive function assessment​

Cognitive function was assessed through two validated domains [19, 20]: Episodic memory measured immediate and delayed recall of 10 words (0–10 each), with participants recalling words immediately after auditory presentation and again after a 10-minute delay. The composite episodic memory score (0–10) averaged both recall performances. Mental intactness was evaluated using the Telephone Interview of Cognitive Status (TICS), comprising orientation (month/date/year/season/weekday; 0–5), visuospatial construction (figure replication; 0–1), and attention (serial-7 subtraction from 100; 0–5), summed as a 0–11 score. Cognition was calculated as the sum of episodic memory and mental intactness scores (0–21), with higher scores indicating better cognitive function.​.

### Covariates

To control for potential confounding factors, this study incorporated covariates across four domains: demographic characteristics (age, gender, marital status, education level), health behaviors (sleep duration, current smoking and drinking status), chronic conditions (hypertension, diabetes, stroke), and psychological health (depressive symptoms)​. Sleep duration, smoking, and drinking status were ascertained through self-reported questionnaires. Chronic conditions were identified through self-reported physician-diagnosed medical histories, and depressive symptoms were evaluated using the 10-item Center for Epidemiologic Studies Depression Scale (CESD-10) [21], with a score of 10 or higher indicating clinically significant symptoms.

### Statistical analysis

The primary outcome was cognitive trajectory patterns. We first constructed a multiple regression model adjusting for age, gender, and education to predict individual cognitive scores. Adjusted Z-scores were calculated as: $\:Z=\frac{Y-\stackrel{-}{Y{\prime\:}}\:}{RMSE}$, where Y represents the observed cognitive score, $\:\stackrel{-}{Y{\prime\:}}$ the predicted mean from the regression model, and RMSE the root mean square error. This standardization approach controlled for demographic confounders to quantify deviations from expected cognitive performance [21].

Using the “gbmt” package in R (v4.4.0), GBTM was applied to Z-scores across five waves with age as the time metric. Binomial models specifying 1–6 trajectory groups were fitted, with optimal group number determined by: (1) minimized Bayesian Information Criterion (BIC), (2) average posterior probability ≥ 70% per group, and (3) minimum group size > 10% of the cohort.

Multinomial logistic regression evaluated headache-cognition trajectory associations, reporting odds ratios (ORs) with 95% confidence intervals (CIs) for two models: Model 1 (unadjusted) and Model 2 (adjusted for demographics, health behaviors, comorbidities, and psychological health). Stratified analyses tested effect modification by gender, education, marital status, smoking, drinking status, hypertension, diabetes, and stroke.

Additionally, sensitivity analysis was conducted by re-running the GBTM using only the first four waves of data. The resulting trajectories were compared to the primary five-wave model to evaluate robustness through consistency in trajectory characteristics (number, shape, and population proportions).​.

​Furthermore, supplementary analysis employed Cox proportional hazards models to assess clinical relevance by examining associations between trajectory groups and incident cognitive impairment.

​Non-response analysis was performed to evaluate potential biases arising from participant exclusion.

All statistical analyses employed two-sided tests with the ​significance level (α = 0.05)​.

## Results

### Baseline characteristics

The study included 2,949 participants with a mean age of 60.19 ± 8.21 years (standard deviation), of whom 58.97% were male. Among this cohort, 361 participants (12.24%) reported headache symptoms at baseline. Comprehensive demographic characteristics, clinical covariates, and cognitive assessment scores are detailed in Table 1.

**Table 1:** Characteristic of the study cohort at baseline

Characteristic	Overall (*N* = 2,949)
Age (years), mean ± SD	60.19 ± 8.21
Gender, n (%)	
Male	1739 (58.97%)
Female	1210 (41.03%)
Education, n (%)	
No formal education	665 (22.55%)
Primary school	788 (26.72%)
Middle school	973 (32.99%)
High school and above	523 (17.73%)
Marital status, n (%)	
Other	190 (6.44%)
Married	2,759 (93.56%)
Current drinker, n (%)	
No	2,087 (70.77%)
Yes	862 (29.23%)
Current smoker, (%)	
No	2,452 (83.15%)
Yes	497 (16.85%)
Hypertension, n (%)	
No	2,249 (76.26%)
Yes	700 (23.74%)
Diabetes, n (%)	
No	2,771 (93.96%)
Yes	178 (6.04%)
Stroke, n (%)	
No	2,904 (98.47%)
Yes	45 (1.53%)
Sleep duration, mean ± SD	6.54 ± 1.65
Depressive symptoms, mean ± SD	7.28 ± 5.77
Headache, n (%)	
No	2,588 (87.76%)
Yes	361 (12.24%)

### Estimated cognitive aging trajectories

We evaluated six group-based trajectory models to capture heterogeneity in global cognitive Z-scores. Table 2 indicates that the three-trajectory model demonstrated an optimal fit with the lowest Bayesian Information Criterion (BIC = 37,724.559). This model satisfied all validity criteria: the average posterior probabilities exceeded 0.7 for all trajectories, indicating high classification accuracy, and each trajectory group comprised more than 10% of the cohort, ensuring adequate sample representation.

**Table 2:** Fit statistics for cognitive function group trajectories in middle-aged and older adults from CHARLS 2011–2020

Fit statistic	Class 1	Class 2	Class 3	Class 4	Class 5	Class 6
AIC*	41445.797	38251.456	37625.776	37626.609	37612.485	37686.144
BIC*	41476.192	38319.841	37724.559	37770.983	37794.853	37906.505
APP^‡^	class1:1.000	class1:0.991	class1:0.988	class1:0.977	class1:0.974	class1:0.982
		class2:0.993	class2:0.988	class2:0.985	class2:0.987	class2:0.938
			class3:0.990	class3:0.980	class3:0.971	class3:0.981
				class4:0.975	class4:0.969	class4:0.956
					class5:0.953	class5:0.965
						class6:0.948
Proportion^¶^	class1:1.000	class1:0.394	class1:0.175	class1:0.104	class1:0.095	class1:0.085
		class2:0.606	class2:0.438	class2:0.224	class2:0.201	class2:0.067
			class3:0.387	class3:0.340	class3:0.294	class3:0.251
				class4:0.333	class4:0.182	class4:0.198
					class5:0.228	class5:0.180
						class6:0.219

*CHARLS* China Health and Retirement Longitudinal Study, *AIC* Akaike’s information criterion, *BIC* Bayesian information criteria, *APP* average posterior probabilities

^*^ A lower absolute value suggests a better model fit

^‡^ A higher value is better (preferably > 0.7 in a class)

^¶^ No less than 10% of total count in a class

**Fig. 2:**
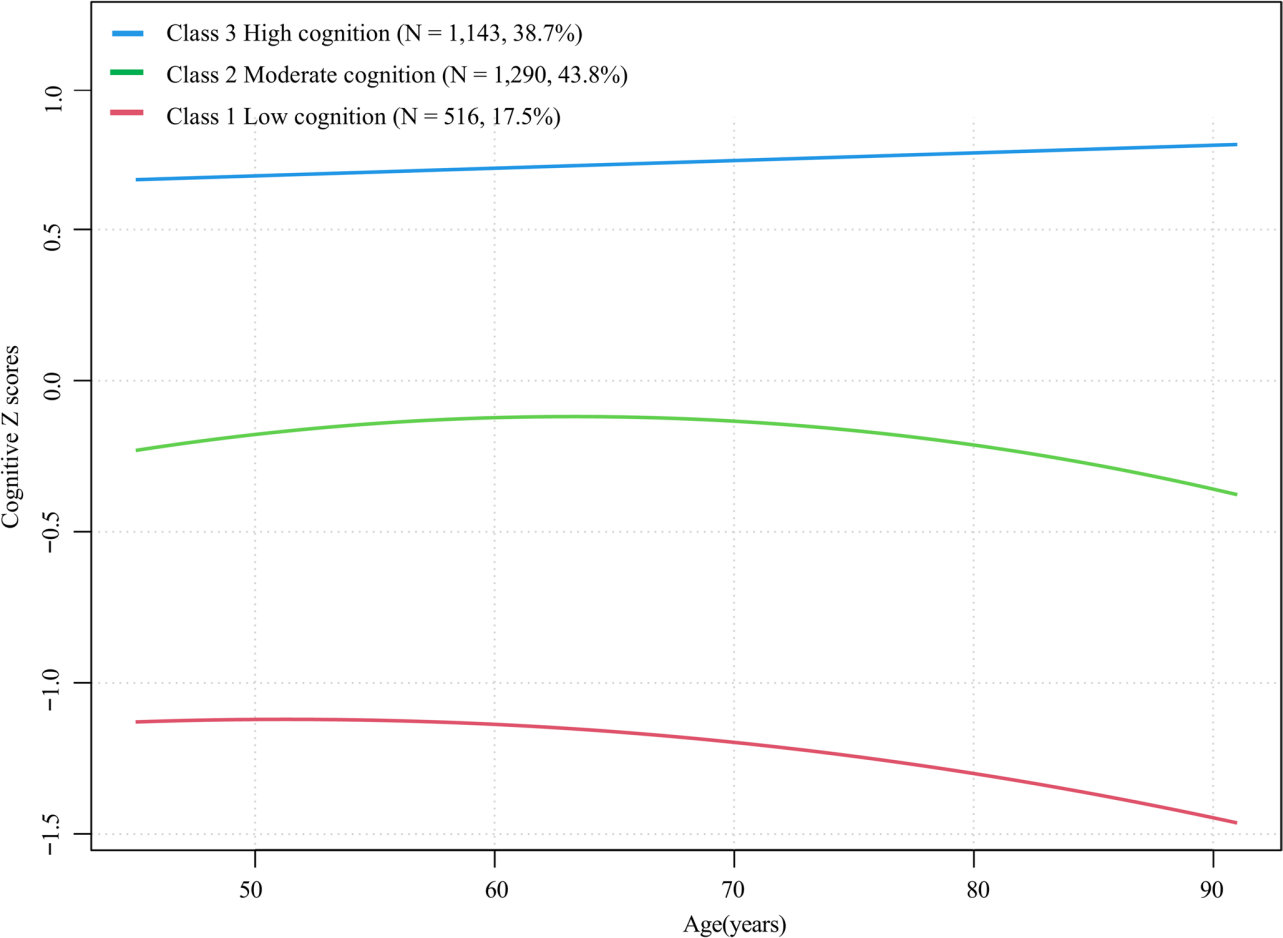
Group-based trajectories of cognitive Z-scores by age among older adults in the CHARLS cohort

Figure 2 illustrates three distinct cognitive trajectories based on longitudinal Z-scores and age at follow-up: Class 1 (“Low Cognition”, *N* = 516, 17.5%), Class 2 (“Medium Cognition”, *N* = 1,290, 43.8%), and Class 3 (“High Cognition”, *N* = 1,143, 38.7%). Maximum likelihood estimates for trajectory parameters are summarized in Table 3.

**Table 3:** The final three-group trajectory model of cognitive Z-scores as a function of age in middle-aged and older adults from CHARLS

Trajectory group	Parameter	Est.	SE	t value	*p* value
Class 1: low cognition (*n* = 516, 17.5%)	Intercept	-1.08E + 00	3.11E-02	-34.728	< 0.001
	Linear (age)	1.89E-03	3.59E-03	0.526	0.600
	Quadratic (age2)	-2.16E-04	9.25E-05	-2.335	0.020
Class 2: medium cognition (*n* = 1,290, 43.8%)	Intercept	-2.39E-01	2.26E-02	-10.594	< 0.001
	Linear (age)	1.28E-02	2.76E-03	4.631	< 0.001
	Quadratic (age2)	-3.32E-04	7.25E-05	-4.415	< 0.001
Class 3: high cognition (*n* = 1,143, 38.7%)	Intercept	6.63E-01	1.17E-02	56.903	< 0.001
	Linear (age)	2.53E-03	6.38E-04	3.956	< 0.001

Est. parameter estimate, SE standard error of parameter estimate

### Cognitive trajectory subgroup characteristics

Table 4 presents the baseline characteristics across cognition trajectory subgroups. Compared with the high cognition group, participants in the low cognition trajectory group were significantly older. They had a higher proportion of male sex, lower education levels, non-marital status, and a higher prevalence of headaches. ​They also had a higher rate of current smoking, shorter sleep duration, and a lower rate of current drinking.​​ Additionally, the low cognition group had significantly higher depressive symptom scores (all *p* < 0.05). In contrast, no significant differences were observed in the prevalence of chronic conditions such as hypertension, diabetes, and stroke between the two groups (all *p* > 0.05).

**Table 4:** Baseline characteristics of middle-aged and older adults according to cognitive trajectory groups in CHARLS

Characteristic	Class 1, low cognition (*N* = 516)	Class 2, medium cognition (*N* = 1,290)	Class 3, high cognition (*N* = 1,143)	*p* value
Age (years), mean ± SD	60.68 ± 8.27	59.87 ± 8.15	60.32 ± 8.24	< 0.001
Gender, n (%)				< 0.001
Male	325 (62.98%)	720 (55.81%)	694 (60.72%)	
Female	191 (37.02%)	570 (44.19%)	449 (39.28%)	
Education, n (%)				< 0.001
No formal education	161 (31.20%)	248 (19.22%)	256 (22.40%)	
Primary school	125 (24.22%)	331 (25.66%)	332 (29.05%)	
Middle school	158 (30.62%)	439 (34.03%)	376 (32.90%)	
High school and above	72 (13.95%)	272 (21.09%)	179 (15.66%)	
Marital status, n (%)				< 0.001
Other	43 (8.33%)	87 (6.74%)	60 (5.25%)	
Married	473 (91.67%)	1,203 (93.26%)	1,083 (94.75%)	
Current drinker, n (%)				0.010
No	375 (72.67%)	898 (69.61%)	814 (71.22%)	
Yes	141 (27.33%)	392 (30.39%)	329 (28.78%)	
Current smoker, (%)				0.017
No	425 (82.36%)	1,064 (82.48%)	963 (84.25%)	
Yes	91 (17.64%)	226 (17.52%)	180 (15.75%)	
Hypertension, n (%)				0.492
No	398 (77.13%)	983 (76.20%)	868 (75.94%)	
Yes	118 (22.87%)	307 (23.80%)	275 (24.06%)	
Diabetes, n (%)				0.169
No	489 (94.77%)	1,210 (93.80%)	1,072 (93.79%)	
Yes	27 (5.23%)	80 (6.20%)	71 (6.21%)	
Stroke, n (%)				0.517
No	509 (98.64%)	1,271 (98.53%)	1,124 (98.34%)	
Yes	7 (1.36%)	19 (1.47%)	19 (1.66%)	
Sleep duration, mean ± SD	6.43 ± 1.83	6.54 ± 1.66	6.59 ± 1.55	< 0.001
Depressive symptoms, mean ± SD	9.43 ± 6.28	7.48 ± 5.79	6.08 ± 5.17	< 0.001
Headache, n (%)				< 0.001
No	422 (81.78%)	1,128 (87.44%)	1,038 (90.81%)	
Yes	94 (18.22%)	162 (12.56%)	105 (9.19%)	

### Relationship between headache and cognitive trajectories​

Analysis of headache-cognition trajectory associations is presented in Table 5. Using the high cognition group as reference, the unadjusted model (Model 1) demonstrated that headache symptoms significantly increased the probability of belonging to both low cognitive trajectories (OR = 2.20, 95% CI: 1.93–2.52; *p* < 0.001) and medium cognitive trajectories (OR = 1.42, 95% CI: 1.26–1.59; *p* < 0.001). After adjustment for covariates including marital status, sleep duration, drinking status, smoking status, hypertension, diabetes, stroke history, and depressive symptoms (Model 2), the association between headache and the low cognition trajectory remained statistically significant (OR = 1.29, 95% CI: 1.11–1.50; *p* < 0.001), while the association with the medium cognition trajectory was also significant (OR = 1.14, 95% CI: 1.01–1.30; *p* = 0.033).

**Table 5:** Multinomial logistic regression analysis of the association between headache and cognitive trajectory groups among middle-aged and older adults in the CHARLS cohort

Variable	low cognition (vs. high cognition)		medium cognition (vs. high cognition)	
	OR (95% CI)*	*p* value	OR (95% CI)*	*p* value
Model 1				
Non-headache	1.00 (reference)		1.00 (reference)	
Headache	2.20(1.93–2.52)	< 0.001	1.42(1.26–1.59)	< 0.001
Model 2				
Non-headache	1.00 (reference)		1.00 (reference)	
Headache	1.29(1.11–1.50)	< 0.001	1.14(1.01–1.30)	0.033

* OR odds ratio, 95% CI 95% confidence intervals

Model 1:​​ unadjusted

​Model 2:​​ adjusted for marital status, sleep duration, drinking status, smoking status, hypertension, diabetes, stroke history, and depressive symptoms

### Subgroup analyses​

**Fig. 3:**
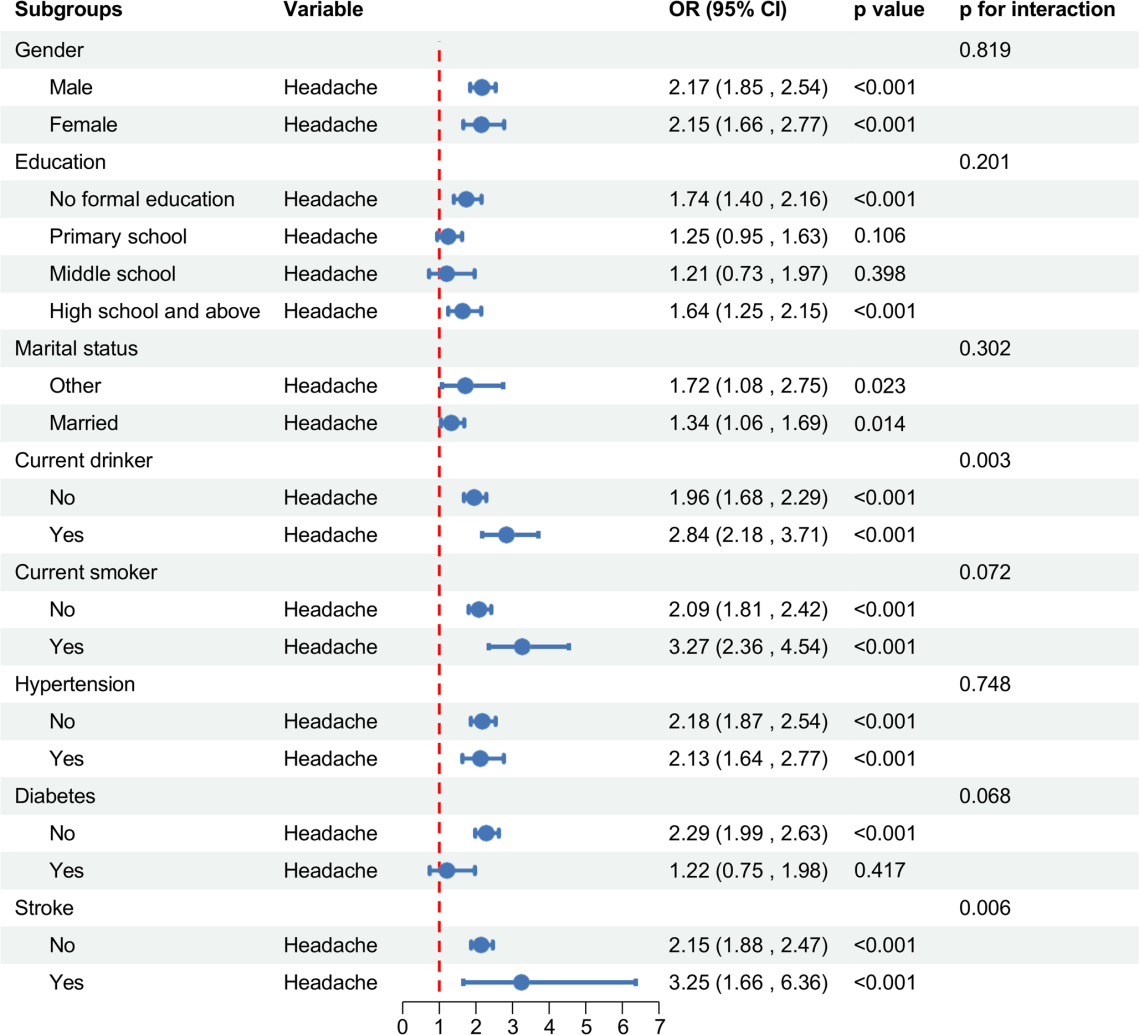
Subgroup-specific associations between headache and low cognitive trajectory group membership (vs. high cognitive group) in the CHARLS cohort

As shown in Fig. 3, headache symptoms significantly increased the probability of belonging to the low cognitive trajectory (*p* < 0.05 for most subgroups). This association persisted across diverse subgroups: males and females; individuals with no formal education and those with high school and above education; married and other marital status individuals; drinkers and non-drinkers; smokers and non-smokers; hypertensive and non-hypertensive individuals; diabetics and non-diabetics; and stroke-free individuals and those with stroke history. Notably, drinking status and stroke history demonstrated significant effect modification (p for interaction < 0.05), with both factors potentiating the association between headache and cognitive decline.

**Fig. 4:**
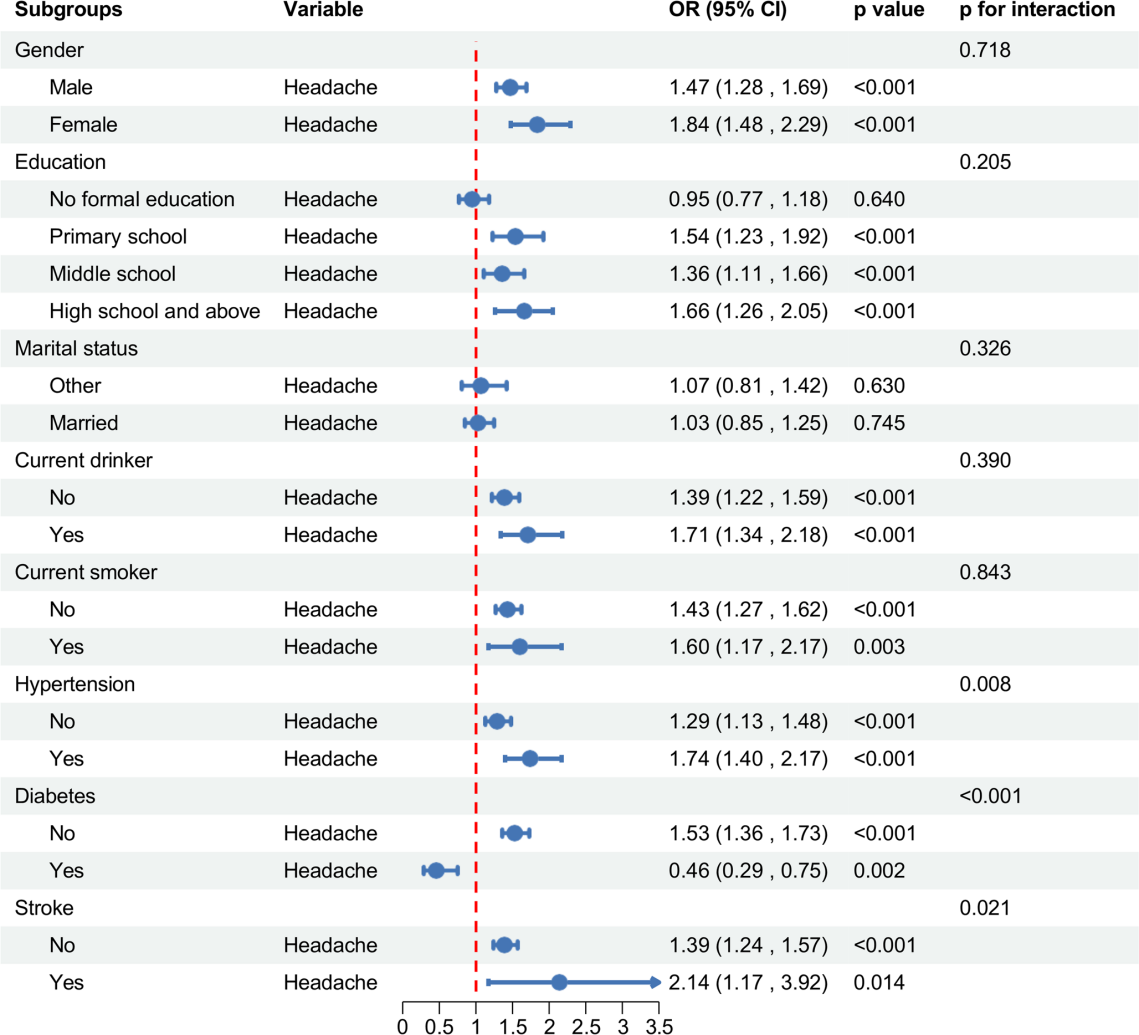
Subgroup-specific associations between headache and medium cognitive trajectory group membership (vs. high cognitive group) in the CHARLS cohort

Figure 4 indicates that headache symptoms significantly elevated the probability of belonging to the medium cognitive trajectory (*p* < 0.05). This association persisted across diverse subgroups: males and females; individuals with primary school, middle school, and high school and above education; drinkers and non-drinkers; smokers and non-smokers; hypertensive and non-hypertensive individuals; diabetics and non-diabetics; and stroke-free individuals and those with stroke history. Notably, hypertension, diabetes, and stroke history demonstrated significant effect modification (p for interaction < 0.05), with hypertension and stroke history potentiating the association between headache and cognitive decline, while diabetes showed a protective effect.

### Sensitivity analyses

To assess the robustness of the model, we compared the trajectories derived from the first four waves of data with those from the full five-wave dataset. The optimal solution for both analyses was a three-trajectory model. Despite the shorter follow-up period, the trajectories identified using the first four waves were broadly comparable to the primary model in their core characteristics. Furthermore, the allocation proportions of participants to each trajectory group remained stable and were similar to those observed in the primary analysis (Figure S1).

Specifically, the four-wave model allocated participants as follows: Class 1 (High cognition), 1,756 participants (35.0%); Class 2 (Moderate cognition), 1,959 participants (39.0%); Class 3 (Low cognition), 1,304 participants (26.0%).

### Supplementary analysis: cognitive trajectories and the risk of cognitive impairment

As presented in Table S1, the supplementary Cox regression analysis revealed a significant gradient in the risk of cognitive impairment across the cognitive trajectory groups. Compared to the low cognitive trajectory group, participants in the medium cognitive trajectory group had a significantly lower risk (adjusted HR = 0.42, 95% CI: 0.27–0.66), while those in the high cognitive trajectory group exhibited the lowest risk (adjusted HR = 0.28, 95% CI: 0.16–0.48).

Figure S2 presents the cumulative incidence curves of severe cognitive impairment by cognitive trajectory group. A significant association was observed, wherein a lower cognitive trajectory was linked to a progressively higher cumulative incidence (Log-rank test, *p* < 0.001).

### Non-response analyses

From the initial CHARLS cohort, 9,113 individuals (75.6%) were excluded from the final analysis due to a lack of follow-up data, incomplete baseline data, or a cognitive score of ≤ 5. Compared to the 2,949 participants included in the study, those who were excluded were significantly older, more likely to be female, and had lower educational attainment. ​In terms of health behaviors, the excluded group had higher rates of current smoking and drinking, as well as shorter sleep duration. They also had poorer health status, characterized by more severe depressive symptoms and a higher prevalence of stroke.​​ However, the prevalence of headache and diabetes did not differ significantly between the groups. As expected, given the exclusion criteria, baseline cognitive function was significantly poorer in the excluded group (Table S2).

## Discussion

By applying GBTM to the CHARLS cohort, we identified three distinct cognitive trajectories: low, medium, and high. Headache symptoms significantly elevated the probability of belonging to low (OR = 1.29; 95% CI: 1.11–1.50) or medium cognitive trajectories (OR = 1.14; 95% CI: 1.01–1.30) after adjusting for demographic characteristics, lifestyle factors, comorbidities, and psychological health. This finding concurs with multinational cohort studies [22–24]. In migraine pathophysiology, cortical spreading depression (CSD)-induced neuronal hyperexcitability [25, 26] and CGRP signaling dysregulation ​converge to disrupt hippocampal-dependent cognitive processes [27].

Furthermore, this study identified drinking status, hypertension status, and stroke as critical effect modifiers that amplify cognitive impairment. Specifically, alcohol activates the TLR4/NF-κB signaling cascade, inducing the release of pro-inflammatory cytokines (IL-1β/TNF-α) by microglia, which exacerbates neuroinflammation and increases cognitive risk [28–30]. Conversely, hypertension impairs cerebral autoregulation, accelerating white matter hyperintensity formation and consequent cognitive deterioration [31–34]. Stroke, as a key modifier, induces cognitive impairment through pathological pathways involving metabolic dysregulation, neuroinflammation, and vascular pathology [35–37], with mechanisms characterized by a complex multifactorial nature.​.

Paradoxically, diabetes exhibited a protective modifying effect on the headache-cognition association in this study (p for interaction < 0.01), contrasting with the established paradigm that diabetes exacerbates cognitive decline [38]. This phenomenon may signify neuroprotective benefits from contemporary diabetes management, particularly through metformin-induced AMPK pathway activation, which suppresses neuroinflammation [39, 40], potentially ameliorating cognitive impairment. Nevertheless, caution is warranted given the limited sample size in the diabetic subgroup, which may affect the magnitude of the effect.

Additionally, this study suggests smoking may exacerbate the headache-cognition association despite a non-significant interaction (p for interaction = 0.072). This potential effect may involve smoking-related neuropathology: smokers show elevated tau protein in cerebrospinal fluid, a biomarker linked to cognitive decline [41], while nicotine disrupts cholinergic neurotransmission in brain regions critical for attention and memory [42]. These mechanisms may synergistically worsen cognitive impairment through interactions with headache pathology.

These mechanistic insights inform stratified interventions, targeting migraine pathophysiology. Preliminary evidence suggests that CGRP monoclonal antibodies may improve cognitive function in migraineurs [44], although their long-term efficacy on cognitive trajectories requires validation in large-scale, randomized controlled trials. For modifiable risk factors, preclinical evidence suggests alcohol abstinence may suppress TLR4-mediated neuroinflammation [43], while intensive blood pressure control mitigates white matter damage progression [44]. Furthermore, reducing nicotine exposure through smoking cessation. Collectively, these interventions confer neuroprotection by reducing neuronal injury through complementary pathways.

​Key limitations of this study should be noted. First, headache was assessed by a single self-report item, lacking details on subtype, frequency, severity, or medication use, which constrains mechanistic interpretation. ​Although we explored an additional item on general pain severity, its formal analysis was precluded by both data availability and conceptual constraints. Second,​​ self-reported data are susceptible to recall bias. ​Third,​​ the findings may have limited generalizability beyond Chinese older adults, necessitating validation in more diverse populations. Future studies should utilize the ICHD-3 criteria and document the use of headache medications.​

## Conclusion

Headache independently predicts adverse cognitive trajectories in middle-aged and older Chinese adults, with alcohol consumption, hypertension, and stroke amplifying risk via modifiable biological pathways, while diabetes mellitus attenuates this risk. Clinically, headache warrants integration into cognitive risk stratification protocols, prioritizing multifactorial interventions targeting alcohol reduction, smoking cessation, ​stroke prevention, and intensive blood pressure control. Future validation in multiethnic cohorts is essential to generalize these strategies.​

## Supplementary Information

Supplementary Material

## Data Availability

Publicly available datasets were analyzed in this study. This data can be found here: http://charls.pku.edu.cn/en/.
