# Supplementary material for: Headache associated with adverse cognitive trajectories in Chinese aging cohort: a group-based trajectory modeling study
Source: Neurol Sci. 2026 Mar 7;47(4):326. doi: 10.1007/s10072-026-08922-8 (PMC12966203; doi:10.1007/s10072-026-08922-8)
Supplement: Supplementary file 1 — Supplementary Material [file 10072_2026_8922_MOESM1_ESM.docx]

**
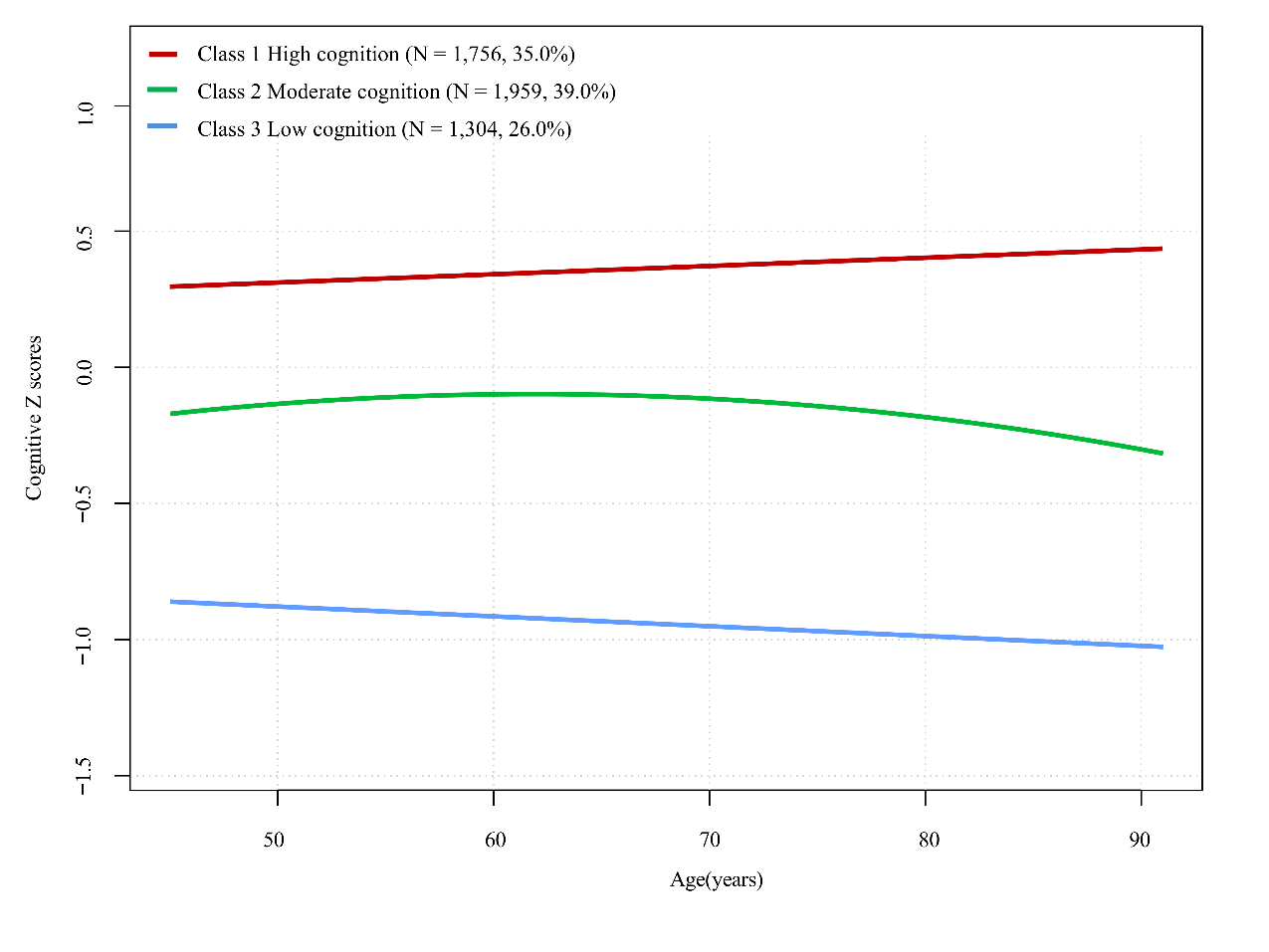
**

#### **Figure S1** Group-based trajectories of cognitive Z-scores by age among older adults in the CHARLS cohort 2011-2018.

**Table S1** Association between cognitive trajectory groups and the risk of cognitive impairment

| **Variable** | **Model 1** | |  | **Model 2** | |
| --- | --- | --- | --- | --- | --- |
|  | **HR (95% CI)*** | **p value** |  | **HR (95% CI)*** | **p value** |
| **Trajectory groups** |  | <0.001 |  |  | <0.001 |
| Low cognition | 1.00 (reference) |  |  | 1.00 (reference) |  |
| Medium cognition | 0.42(0.27-0.65) |  |  | 0.42(0.27-0.66) |  |
| High cognition | 0.27(0.16-0.45) |  |  | 0.28(0.16-0.48) |  |
| **P for trend** |  | <0.001 |  |  | <0.001 |

* HR Hazard ratio, 95% CI 95% confidence intervals

Model 1:​​ unadjusted

​Model 2:​​ adjusted for marital status, sleep duration, drinking status, smoking status, hypertension, diabetes, stroke history, and depressive symptoms


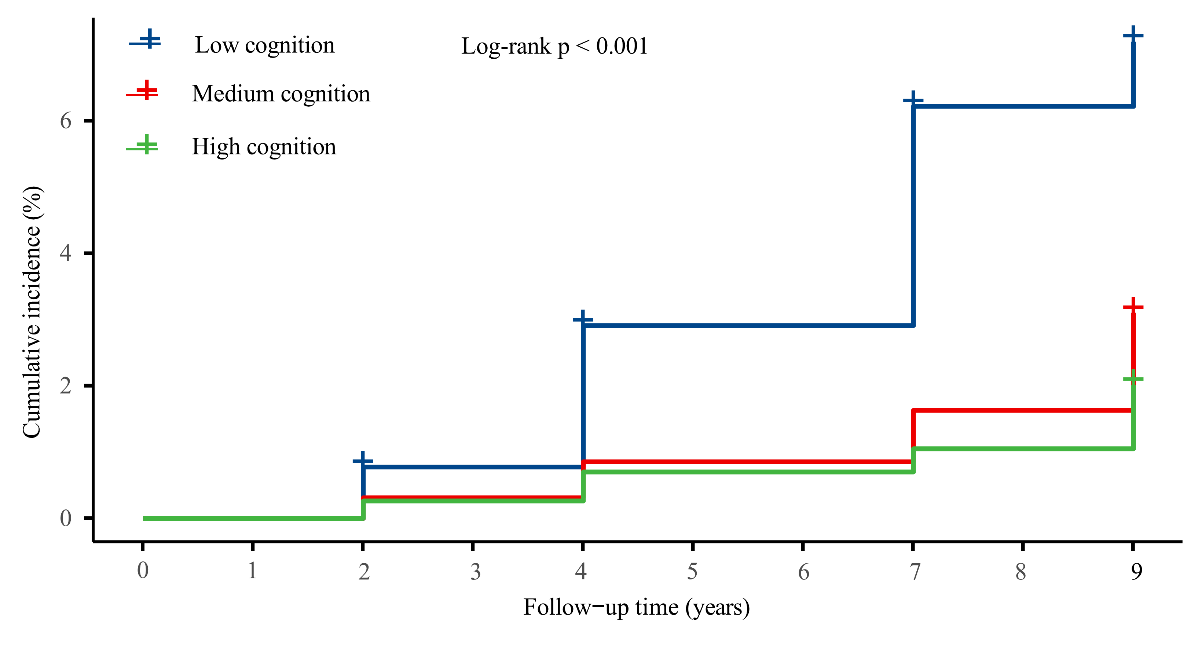


**Figure S2** Kaplan–Meier survival curves for cumulative incidence of cognitive impairment by cognitive trajectory group.​

**Table S2** Baseline characteristics of the final analytical cohort versus those excluded from the CHARLS study

| **Characteristic** | **Non-responders**  **(N = 9113)** | **Analytical cohort​**  **(N = 2949)** | **p value** |
| --- | --- | --- | --- |
| Age (years), mean ± SD | 59.52 ± 9.39 | 55.79 ± 7.54 | <0.001 |
| Gender, n (%) |  |  | <0.001 |
| Male | 4,134.0 (45.4%) | 1,739.0 (59.0%) |  |
| Female | 4,979.0 (54.6%) | 1,210.0 (41.0%) |  |
| Education, n (%) |  |  | <0.001 |
| No formal education | 4,003.0 (43.9%) | 665.0 (22.6%) |  |
| Primary school | 1,985.0 (21.8%) | 788.0 (26.7%) |  |
| Middle school | 1,884.0 (20.7%) | 973.0 (33.0%) |  |
| High school and above | 1,241.0 (13.6%) | 523.0 (17.7%) |  |
| Marital status, n (%) |  |  | <0.001 |
| Other | 1,127.0 (12.4%) | 190.0 (6.4%) |  |
| Married | 7,986.0 (87.6%) | 2,759.0 (93.6%) |  |
| Current drinker, n (%) |  |  | <0.001 |
| No | 5,782.0 (63.4%) | 2,087.0 (70.8%) |  |
| Yes | 3,331.0 (36.6%) | 862.0 (29.2%) |  |
| Current smoker, (%) |  |  | <0.001 |
| No | 5,706.0 (62.6%) | 2,452.0 (83.1%) |  |
| Yes | 3,407.0 (37.4%) | 497.0 (16.9%) |  |
| Hypertension, n (%) |  |  | <0.001 |
| No | 6,657.0 (73.0%) | 2,249.0 (76.3%) |  |
| Yes | 2,456.0 (27.0%) | 700.0 (23.7%) |  |
| Diabetes, n (%) |  |  | 0.311 |
| No | 8,515.0 (93.4%) | 2,771.0 (94.0%) |  |
| Yes | 598.0 (6.6%) | 178.0 (6.0%) |  |
| Stroke, n (%) |  |  | <0.001 |
| No | 8,862.0 (97.2%) | 2,904.0 (98.5%) |  |
| Yes | 251.0 (2.8%) | 45.0 (1.5%) |  |
| Sleep duration | 6.37 ± 1.86 | 6.54 ± 1.65 | <0.001 |
| Depressive symptoms | 8.15 ± 6.26 | 7.28 ± 5.77 | <0.001 |
| Headache, n (%) |  |  | 0.917 |
| No | 7985 (87.83%) | 2607 (87.75%) |  |
| Yes | 1106 (12.17%) | 364 (12.25%) |  |
| Cognitive Score | 11.65 ± 3.65 | 13.34 ± 2.70 | <0.001 |
